# The prognostic significance of malnutrition in older adult patients with acute ischemic stroke

**DOI:** 10.3389/fnut.2025.1529754

**Published:** 2025-01-31

**Authors:** Tian-Tian Jiang, Xing-Yu Zhu, Yan-Wei Yin, Hong-Jin Liu, Guang-Yun Zhang

**Affiliations:** ^1^Graduate School of Hebei North University, Zhangjiakou, Hebei, China; ^2^Neurology, Air Force Medical Center, Chinese People's Liberation Army, Beijing, China; ^3^Accreditation Section, Air Force Medical Center, Chinese People's Liberation Army, Beijing, China

**Keywords:** acute ischemic stroke, prognostic nutrition index, Naples prognostic score, controlling nutritional status, geriatric nutritional risk index, hemoglobin, albumin, lymphocyte and platelet score

## Abstract

**Background:**

Malnutrition is associated with an unfavorable prognosis; however, malnutrition in hospitalized patients is frequently overlooked by clinicians. This highlights the importance of accurately assessing nutritional status and providing appropriate nutritional supplementation. The most appropriate nutritional assessment tool for predicting the short-term prognosis of older adult patients with Acute Ischemic Stroke (AIS) was identified from five nutritional assessment tools, including the Prognostic Nutrition Index (PNI), the Hemoglobin, Albumin, Lymphocyte and Platelet (HALP) Score, the Naples Prognostic Score (NPS), the Geriatric Nutritional Risk Index (GNRI), and the Controlling Nutritional Status (CONUT) Score.

**Methods:**

A total of 585 older adult patients with Acute Ischemic Stroke (AIS) were retrospectively analyzed and divided into two groups according to the modified Rankin Scale (mRS) score. The first group, comprising 111 cases, was classified as having a poor prognosis (mRS score > 2), while the second group, consisting of 391 cases, was classified as having a good prognosis (mRS score ≤ 2). A total of five nutritional assessment tools, including PNI, HALP Score, NPS, GNRI, and CONUT, were employed to evaluate the nutritional status of older adult patients with AIS and for the analysis of the relationship between nutritional status and prognosis. The incremental value of five nutritional assessment tools in predicting patient prognosis was compared by means of the Integrated Discriminant Improvement Index (IDI) and the Net Reclassification Index (NRI). The efficacy of each nutritional assessment tool in forecasting the incidence of unfavorable outcomes in older adult patients with AIS within a one-year timeframe was evaluated by utilizing the area under the receiver operating characteristic curve (AUC), calibration curves, and decision analysis curves. Comparative analyses were also conducted.

**Result:**

Among the five nutritional assessment tools, the PNI (AUC: 0.619, 95% CI: 0.560–0.679, *p* < 0.001) and HALP score (AUC: 0.612, 95% CI: 0.552–0.672, p < 0.001) demonstrated a significantly greater area under the ROC curve (AUC) compared to the NPS (AUC: 0.597, 95% CI: 0.536–0.658, *p* = 0.002), CONUT score (AUC: 0.582, 95% CI: 0.520–0.644, *p* = 0.009), and GNRI (AUC: 0.590, 95% CI: 0.529–0.651, *p* < 0.001). When compared to BMI, PNI exhibited a more pronounced improvement in the integrated discrimination index (IDI: 0.0203, *p* = 0.0061). Similarly, the net reclassification index (NRI) also showed a significant improvement (NRI: 0.2422, *p* = 0.024), indicating the superior performance of PNI in risk stratification.

**Conclusion:**

Among the five types of nutritional assessment tools employed in this study, the PNI was the most effective at predicting a poor prognosis at one year in older adult patients with AIS.

## 1 Introduction

Acute Ischemic Stroke (AIS) is a cerebrovascular disorder characterized by the acute reduction of blood flow to the brain, caused by the formation of a thrombus or blood clot within a cerebral artery. It is the leading cause of disability and death worldwide (1). Malnutrition has been demonstrated to have a detrimental impact on the prognosis of stroke patients. It is associated with an increased length of hospitalization due to complications, mortality, and a poor neurological prognosis in acute stroke patients (2). However, malnutrition in hospitalized patients is frequently overlooked by clinicians, underscoring the importance of accurately assessing nutritional status and providing appropriate nutritional supplements. As the global population continues to age, the cohorts of individuals aged 65 and above, and in particular those aged 80 and above, are set to experience the most rapid growth (3). Consequently, the older adult population is expanding, which will lead to a rise in AIS cases among this demographic. The search for a nutritional assessment tool that can more effectively evaluate the nutritional status of older adult patients with AIS is of paramount importance in the context of older adults, who frequently encounter nutritional challenges associated with organ and body function decline, underlying diseases, and poor dietary habits.

The Prognostic Nutritional Index (PNI), Hemoglobin, Albumin, Lymphocyte and Platelet (HALP) Score, Naples Prognostic Score (NPS), Geriatric Nutritional Risk Index (GNRI), and Controlling Nutritional Status (CONUT) score can be calculated rapidly and objectively based on the analysis of blood parameters and the measurement of height and weight. These scores can be rapidly calculated based on blood parameters and height and weight and can be employed to indicate the nutritional status of patients straightforwardly and objectively (4, 5). The PNI is a nutritional assessment tool based on serum albumin levels and lymphocyte counts. Initially developed for the evaluation of immunological and nutritional aspects in patients undergoing gastrointestinal surgery, its applications have since expanded. Subsequently, it has been employed in the assessment of a multitude of other conditions, including cancer, chronic kidney disease, and cardiovascular disease (6–8). The HALP score is a combination of four common clinical indicators that are now widely used in the prognostic assessment of various tumors, cardiovascular diseases, inflammation, fibrosis, and other conditions. The HALP score was initially developed by Prof. Hu Jian Kun of the Gastric Cancer Centre of West China Hospital in 2015. It integrates three key indicators: peripheral inflammation, nutritional status, and immune status. This integration reduces the limitations of a single indicator and makes it an economically viable option for use in clinical practice. Prior research has indicated that HALP Score is an effective prognostic indicator for patients with diverse tumor types and is a reliable predictor of dyslipidemia (9–11). The NPS, devised by the Italian scholar Galizia, is based on the levels of Neutrophil-to-Lymphocyte Ratio (NLR), Lymphocyte-to-Monocyte Ratio (LMR), albumin, and total cholesterol. This integrated approach allows for the simultaneous assessment of systemic inflammation, nutritional status, and immune function (12). The GNRI is a composite index that considers both serum albumin levels and body weight. It is a widely utilized tool for assessing the nutritional status of hospitalized older adults (13). The CONUT score, developed by Ulibarri et al. in 2005, serves as a screening tool for assessing the nutritional status of hospitalized patients (14). It is a composite index based on total lymphocyte count, total cholesterol levels, and serum albumin, providing a comprehensive evaluation of a patient’s nutritional condition.

Nevertheless, the question of which score is more effective in predicting a poor prognosis at 1 year in older adult patients with AIS remains unanswered. Accordingly, we employed five objective nutritional status assessment tools to predict the prognosis of older adult patients with AIS within 1 year and to ascertain which nutritional assessment tool is more suitable for the nutritional assessment of older adult patients with AIS. The objective was to inform clinicians of the most appropriate nutritional assessment tool for use in early clinical decision-making and intervention.

## 2 Methods

### 2.1 Study design and selection of patients

**Figure 1:**
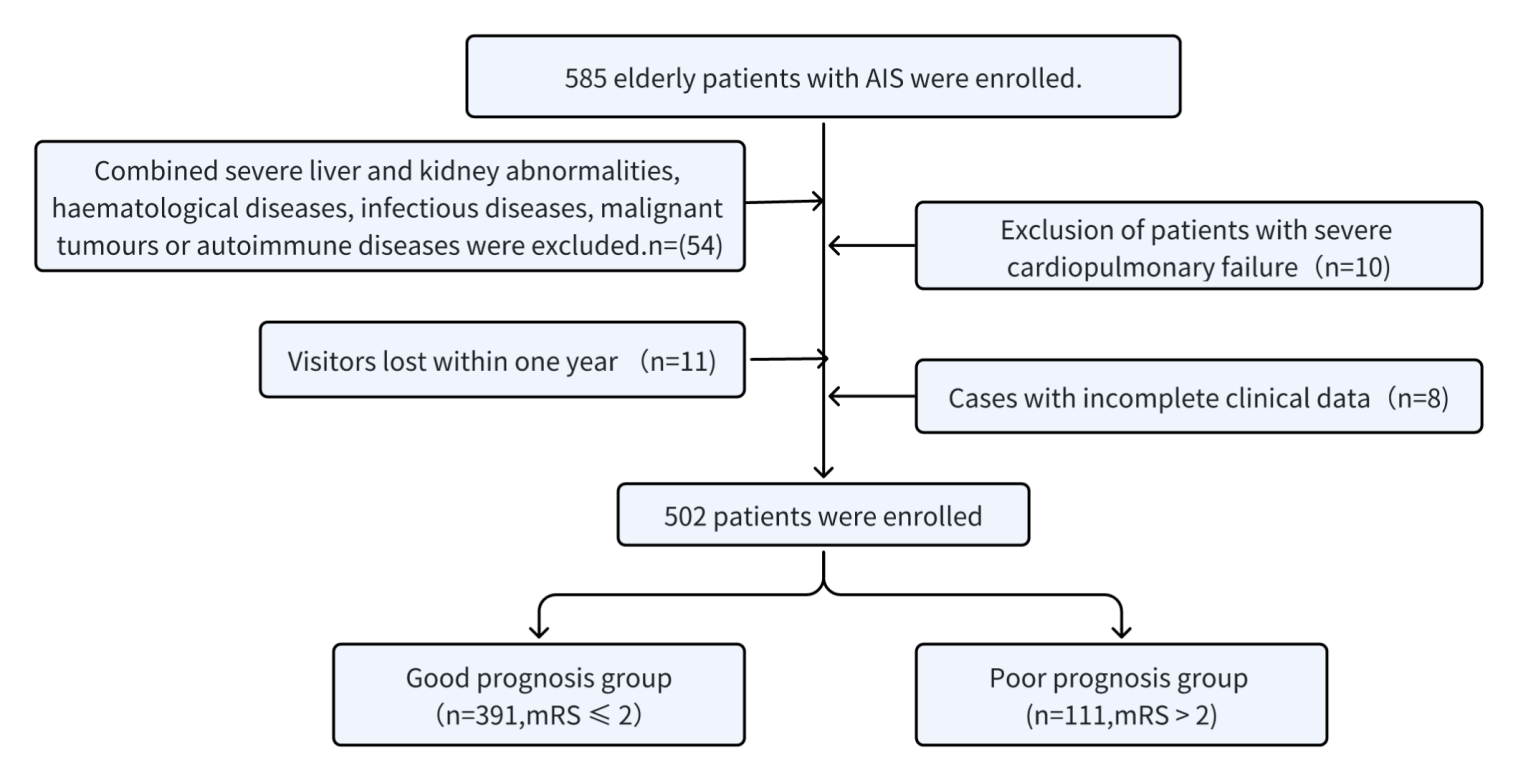
Flow diagram indicating study population.

This was a retrospective observational study based on data from the Chinese People’s Liberation Army Air Force Medical Centre. The study included 585 consecutive older adult patients with a diagnosis of AIS admitted to the Department of Neurology from February 2022 to August 2023. A total of 502 patients were included based on the inclusion and exclusion criteria. Patients were stratified according to the modified Rankin Scale (mRS) scores into a poor prognosis group (n = 111, mRS > 2) and a favorable prognosis group (*n* = 391, mRS ≤ 2). The following criteria were used to determine eligibility for inclusion in the study: To be eligible for inclusion in the study, participants must meet the following criteria: (1) age ≥ 60 years; (2) complete medical records; (3) meet the diagnostic criteria set out in the Chinese Guidelines for the Diagnosis and Treatment of Acute Ischaemic Stroke 2018 (15). The following criteria were used to exclude participants from the study: (1) Pre-onset mRS score > 2; (2) absence of laboratory data within 24 h of admission; (3) lack of mRS score data one year after hospitalization; (4) neurological disorders such as acute traumatic brain injury and dementia with intracranial malignant tumors; (5) hematological diseases such as severe liver and renal dysfunction; (6) Severe cardiorespiratory insufficiency; (7) A history of severe disability or post-stroke sequelae that interfere with the National Institutes of Health Stroke Scale (NIHSS) score. The study was approved by the Ethics Committee of the Chinese People’s Liberation Army Air Force Medical Centre. The study was conducted in accordance with the Declaration of Helsinki and related guidelines and regulations, and all participants provided informed consent to participate. The study’s flow chart is presented in Figure 1.

### 2.2 Research methodology: basic information collection

The general clinical data of the patient were collected, including the patient’s basic clinical characteristics, namely age, sex, height, weight, admission blood pressure, and smoking history. The patient’s past medical history included a history of hypertension, diabetes mellitus, hyperlipidemia, coronary heart disease, atrial fibrillation, and other relevant conditions. Additional tests were conducted to obtain further information, including a complete blood count (leucocytes, neutrophils, lymphocytes, hemoglobin, platelets, and ultrasensitive C-reactive protein), liver function tests, renal function tests, and lipid profiles. All patients were observed for one year, with short-term outcomes assessed via outpatient visits or telephone interviews using the mRS. An mRS score of >2 was considered indicative of a poor prognosis, while an mRS score of ≤2 was deemed to indicate a favorable outcome.

### 2.3 Nutritional status assessment tools

The PNI was calculated using the following formula: PNI = albumin (g/L) + 5 × lymphocytes (×10^9^/L) (16). A score of >38 indicated normal nutritional status, while scores of 35–38 and < 35 reflected moderate and severe malnutrition, respectively. The HALP score was determined using the formula: HALP = hemoglobin (g/L) × albumin (g/L) × lymphocytes (/L)/platelets (/L) (9). The NPS is calculated as the sum of the plasma albumin level, total cholesterol level, NLR, and LMR scores. The definition and grading criteria of NPS, as proposed by Galizia et al. (12), are presented in Table 1. The GNRI was calculated using the formula: GNRI = 1.489 × serum albumin (g/L) + 41.7 × (body weight (kg)/ideal body weight (kg)). Based on GNRI classification, scores were categorized as follows: ≥98: no risk of malnutrition; 92–98: mild risk of malnutrition; 82–92: moderate risk of malnutrition; <82: severe risk of malnutrition. The CONUT score, developed by Ulibarri et al. in 2005, serves as a screening tool to assess the nutritional status of hospitalized patients (14). The score is calculated based on the patient’s serum albumin, total cholesterol, and total lymphocyte count, with the scoring guidelines detailed in Table 2. The scores for each of the three indicators are summed to obtain a total score (range 0–12), with higher total scores indicating poorer nutritional status. The total score is classified as follows: 0–1 is classified as nutritionally normal, 2–4 is classified as mildly malnourished, 5–8 is classified as moderately malnourished, and 9–12 is classified as severely malnourished.

**Table 1:** The Grading Standard of NPS.

Grade(point)	Albumin (g/dl)	TC (mg/dL)	NLR	LMR
0	≥ 4	>180	≤ 2.96	>4.44
1	< 4	≤ 180	> 2.96	≤ 4.44

TC, total cholesterol; NLR, Neutrophil-lymphocyte ratio; LMR, Lymphocyte-Monocyte Ratio.

**Table 2:** The Grading Standard of COUNT.

Parameters	Normal	Light	Moderate	Severe
Serum albumin (g/dL)	≥ 3.5	3.00–3.49	2.50–2.99	<2.50
Score	0	2	4	6
Total cholesterol (mg/dL)	>180	140–180	100–139	<100
Score	0	1	2	3
Total lymphocyte count	>1.6 × 10^9^/L	(1.2–1.6) × 10^9^/L	(0.8–1.1) × 10^9^/L	<0.8 × 10^9^/L
Score	0	1	2	3
COUNT	0–1	2–4	5–8	9–12
Score	Normal	Light	Moderate	Severe

### 2.4 Statistical methods

The statistical analyses were conducted using R (4.2.3) and SPSS 27.0 software. The normally distributed measurements were expressed as the mean ± standard deviation (𝑥̅ ± 𝑠), and the comparisons were made using the t-test. The non-normally distributed measurements were expressed as the median [M(Q1, Q3)], and the comparisons were made using the Mann–Whitney U-test. Finally, the counts were expressed as percentages, which were then subjected to a χ2 test. The objective was to evaluate the efficacy of each nutritional status assessment tool in predicting the prognosis for older adult patients with AIS at 1 year. To this end, the AUC of subjects’ work characteristics (ROC) was plotted using SPSS, to assess the sensitivity and specificity of the five nutritional status assessment tools for poor prognosis in older adult patients with AIS. A *p*-value of less than 0.05 was used as the criterion for a statistically significant difference. The net reclassification index (NRI) was calculated using the ‘survival’ and ‘prices’ packages in the R software. The integrated discriminant improvement index (IDI) was calculated using the ‘PredictABEL’, ‘survival’, and ‘rms’ packages to calculate the IDI, which was used to measure the incremental value of the five nutrient assessment tools. The ‘xgboost’ and ‘randomForest’ packages in R were employed to construct SHAP summary plots for several nutritional assessment tools, to analyze the importance of the characteristics of the variables and the effects of the characteristics. Calibration curves, clinical decision curves, and clinical impact curves were constructed using the ‘regplot’ and ‘rmda’ packages in R software to determine the differentiation, calibration, and impact of the five nutritional indicators in predicting whether a poor prognosis would occur within one year in older adult patients with AIS. A *p*-value of less than 0.05 indicates that the observed difference is statistically significant.

## 3 Results

### 3.1 Comparison of general clinical data between the good prognosis group and the poor prognosis group

Of the 502 enrolled patients, 111 were classified into the poor prognosis group and 391 into the good prognosis group based on the mRS score. The poor prognosis group exhibited significantly elevated levels of age, leukocyte count, neutrophil count, C-reactive protein, and NLR compared to the good prognosis group. Conversely, the good prognosis group demonstrated significantly reduced levels of PNI, GNRI, and HALP Score compared to the poor prognosis group, with a statistically significant difference (*p* < 0.05, Table 3).

**Table 3:** Comparison of general clinical data between the good prognosis group and the poor prognosis group.

Variables	The good prognosis group (n = 391)	The poor prognosis group (n = 111)	*P*
Gender (male, %)	225 (57)	59 (53)	0.570
Age [years old, M (Q1, Q3)]	69.00 (65.00, 77.50)	75.00 (68.50, 83.50)	< 0.001
Systolic blood pressure (mmHg, 𝑥̅±𝑠)	145.08 ± 20.99	149.55 ± 21.58	0.054
Height [m, M (Q1, Q3)]	1.66 (1.60, 1.71)	1.64 (1.60, 1.70)	0.181
Body weight [Kg, M (Q1, Q3)]	67.00 (60.00, 75.00)	65.00 (60.69, 70.00)	0.089
BMI [Kg/m^2^, M (Q1, Q3)]	24.49 (22.58, 26.49)	23.66 (22.48, 26.03)	0.130
Smoking, n (%)	151 (39)	31 (28)	0.050
Atrial fibrillation, n (%)	23 (6)	13 (12)	0.058
White blood cell count [×10^9^/L, M (Q1, Q3)]	6.36 (5.28, 7.75)	7.47 (5.72, 8.92)	< 0.001
Hemoglobin [g/L, M (Q1, Q3)]	134 (124, 144)	134 (122, 144)	0.585
Platelet count [×10^9^/L, M (Q1, Q3)]	213.00 (176.50, 256.50)	212.00 (181.50, 253.50)	0.899
Neutral particle count [×10^9^/L, M (Q1, Q3)]	4.14 (3.09, 5.22)	4.90 (3.76, 6.53)	< 0.001
Lymphocyte [×10^9^/L, M (Q1, Q3)]	1.60 (1.23, 2.04)	1.37 (1.04, 1.77)	0.002
Hs-CRP [mg/L, M (Q1, Q3)]	1.51 (0.55, 3.48)	2.72 (1.38, 8.72)	< 0.001
Creatinine [μmol/L, M (Q1, Q3)]	68.00 (57.40, 82.00)	65.00 (52.65, 83.35)	0.186
Uric acid [μmol/L, M (Q1, Q3)]	306.00 (254.24, 358.50)	313.40 (236.00, 389.50)	0.804
Albumin [g/L, M (Q1, Q3)]	41.60 (39.02, 44.20)	40.54 (37.67, 43.15)	0.003
Total cholesterol, [mmol/L, M (Q1, Q3)]	4.29 (3.52, 5.23)	4.18 (3.36, 5.12)	0.393
Triglyceride [mmol/L, M(Q1,Q3)]	1.29 (0.97, 1.75)	1.21 (0.90, 1.65)	0.251
HDL [mmol/L, M (Q1, Q3)]	1.08 (0.93, 1.25)	1.06 (0.90, 1.29)	0.496
LDL [mmol/L, M (Q1, Q3)]	2.36 (1.80, 3.06)	2.36 (1.71, 3.00)	0.605
PNI [M (Q1, Q3)]	49.86 (46.41, 53.06)	47.43 (44.30, 50.98)	< 0.001
GNRI [M (Q1, Q3)]	109.47 (103.76, 115.08)	106.51 (100.87, 111.98)	0.004
NLR [M (Q1, Q3)]	2.52 (1.78, 3.47)	3.24 (2.32, 5.41)	< 0.001
LMR [M (Q1, Q3)]	3.88 (2.91, 5.18)	3.34 (2.26, 4.37)	< 0.001
NPS, n (%)			< 0.001
0	43 (11)	6 (5)	
1	99 (25)	20 (18)	
2	117 (30)	26 (23)	
3	99 (25)	35 (32)	
4	33 (8)	24 (22)	
CONUT Score [M (Q1, Q3)]	2 (1, 3)	2 (1, 3)	< 0.001
HALP Score [M (Q1, Q3)]	41.60 (31.13, 55.19)	34.46 (25.50, 47.45)	< 0.001

BMI, Body Mass Index; HDL, High Density Lipoprotein; LDL, Low Density Lipoprotein; PNI, Prognostic Nutritional Index; HALP, Hemoglobin, Albumin, Lymphocyte, and Platelet Score; NPS, Naples Prognostic Score; GNRI, Geriatric Nutritional Risk Index; CONUT, Controlling Nutritional Status; NLR, Neutrophil-Lymphocyte Ratio; LMR, Lymphocyte Monocyte Ratio.

**Figure 2:**
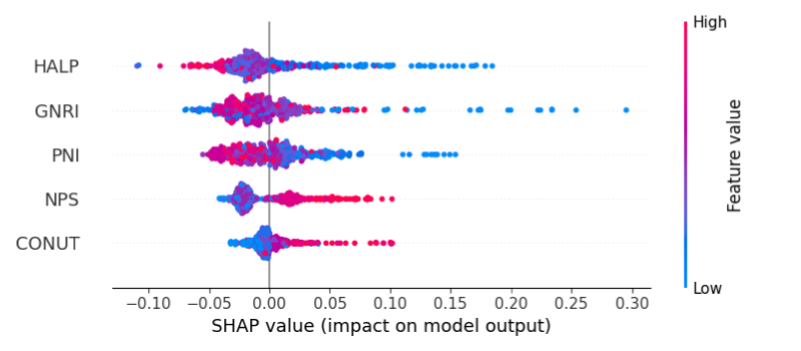
Illustrates the SHAP summary plot, which demonstrates the application of machine learning to feature selection.

In this study, we constructed a SHAP summary plot using machine learning with five nutritional assessment tools as variables. The aim was to predict outcome events using whether older adult AIS patients had a poor prognosis at 1 year as an outcome. The results are presented in Figure 2. The rows in Figure 2 represent the features, with the horizontal coordinate indicating the SHA*p* value. The features are ordered according to the mean absolute value of SHAP, which represents the most important feature of the model. The width of the area indicates the number of samples that are clustered together. The color of a dot represents the value of the feature associated with the sample. Redder dots indicate larger values, while bluer dots indicate smaller values. As illustrated in Figure 2, a reduction in HALP Score, GNRI, and PNI values is associated with an increased likelihood of poor prognosis, whereas an elevation in NPS and CONUT Score values is linked to a heightened probability of poor prognosis.

**Figure 3:**
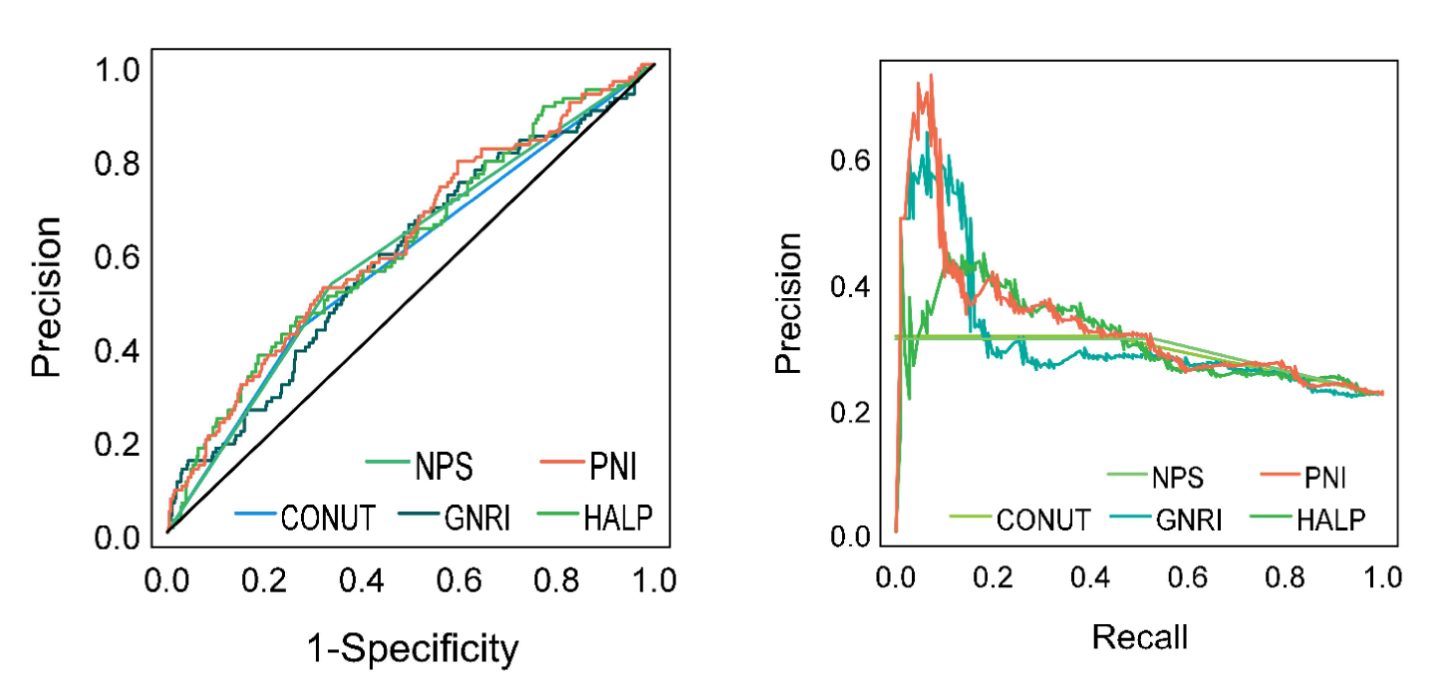
ROC and precision-recall curves for five nutritional assessment tools to predict whether older adult AIS patients have a poor prognosis at 1 year.

In this study, five types of nutritional assessment tools were employed to ascertain whether older adult patients with AIS would have an unfavorable prognosis at one year. ROC curves were constructed to evaluate the predictive efficacy of these tools (Table 4; Figure 3). With regard to the AUC, the PNI demonstrated a higher AUC (0.619, 95% CI: 0.560–0.679, *p* < 0.001) than the remaining four nutritional assessment tools. As illustrated in Figure 3, the precision-recall curve demonstrates that the PNI exhibits superior recall characteristics in comparison to the other nutritional indicators. The optimal cut-off values for PNI, HALP Score, NPS, GNRI, and CONUT Score were determined using the established Jordon’s index. The five nutritional assessment tools were divided into two groups according to the optimal cut-off values, resulting in the following categories: high and low PNI, high and low HALP Score, high and low NPS, high and low GNRI, and high and low CONUT Score.

**Table 4:** Comparison of AUCs of nutritional assessment tools.

Models	AUC	95% CI	*P* value for AUCs	Cut-off value
PNI	0.619	0.560–0.679	<0.001	47.57
HALP Score	0.612	0.552–0.672	<0.001	31.72
NPS	0.597	0.536–0.658	0.002	2.50
GNRI	0.590	0.529–0.651	0.004	107.25
CONUT Score	0.582	0.520–0.644	0.009	2.50

### 3.2 A one-way logistic regression analysis was conducted

A one-way logistic regression analysis was conducted to investigate the relationship between the prognosis of older adult patients with AIS at one year and five nutritional assessment tools, including PNI, HALP Score, NPS, GNRI, and CONUT Score. The dependent variable was defined as the poor prognosis of older adult patients with AIS at one year, while the independent variables were the aforementioned nutritional assessment tools. The ratio of odds (OR) and 95% confidence intervals (CI) were calculated to determine the strength of the relationship between the two variables. A positive regression coefficient and an OR value greater than 1 indicate that the factor is a risk factor for the outcome. Conversely, a negative regression coefficient and an OR value less than 1 suggest that the factor is a protective factor. An OR value of 1 indicates that the factor does not play a role in the occurrence of the disease. Table 5 illustrates the presence of low PNI, low HALP Score, high NPS, low GNRI, and high CONUT Score, which can be considered risk factors. The relative risk (RR) indicates the degree of influence of the control factors on the occurrence of the event outcome. As can be seen from Table 5, there is a 1.425-fold increased likelihood of a poor prognosis occurring in cases where the PNI is low, compared to instances where the PNI is high.

**Table 5:** Results of one-way logistic regression analysis for poor prognosis.

Characteristic	OR	95%CI	*P* value	Relative risk	95% CI
Low PNI	2.329	1.517–3.576	<0.001	1.425	1.159–1.751
Low HALP Score	2.346	1.517–3.626	<0.001	1.358	1.132–1.628
High NPS	2.226	1.452–3.414	<0.001	1.414	1.146–1.745
Low GNRI	1.927	1.258–2.950	0.003	1.367	1.093–1.711
High CONUT Score	2.075	1.340–3.213	0.001	1.289	1.084–1.532

### 3.3 Five nutritional assessment tools versus BMI

The NRI represents a principal method for evaluating the precision of a predictive model. A positive NRI value indicates that the new model outperforms the old model in terms of predictive efficacy. Conversely, a negative NRI value implies that the new model underperforms the old model. The IDI is a metric that assesses the change in the discrepancy in predictive probability between the two models. It is derived from the predictive probability of each individual in the disease model. In general, higher values of the IDI indicate that the new model demonstrates a greater degree of superiority in predictive ability. If the IDI is greater than zero, this indicates a positive improvement; if the IDI is less than zero, it indicates a negative improvement; and when the IDI is equal to zero, it indicates that the new model exhibits no improvement. As can be observed from Table 6, the IDI value for PNI is 0.0203 and the NRI value is 0.2422. Furthermore, the 95% confidence interval does not cover 0, and the *p*-value is less than 0.05. These findings collectively suggest that PNI is a superior predictor of BMI in determining whether an older adult patient with AIS will experience a poor prognosis within one year. The NPS and CONUT Score were similarly effective in predicting a poor prognosis for older patients with AIS at one year when compared to the BMI.

**Table 6:** Comparison of five malnutrition assessment tools with BMI.

Models	IDI			NRI		
	Absolute IDI	95%CI	*P* value	Total NRI	95% CI	*P* value
PNI	0.0203	0.0058–0.0348	0.0061	0.2422	0.0326–0.4519	0.0240
HALP Score	0.0177	0.0061–0.0284	0.0029	0.1611	−0.0468-0.3689	0.1288
NPS	0.0229	0.0088–0.0370	0.0015	0.3428	0.1360–0.5496	0.0012
GNRI	0.0093	0.0009–0.0177	0.0292	0.1214	−0.0890-0.3318	0.2580
CONUT Score	0.0203	0.0038–0.0369	0.0162	0.2186	0.0090–0.4282	0.0409

PNI, Prognostic Nutritional Index; HALP Score, Hemoglobin, Albumin, Lymphocyte and Platelet Score; NPS, Naples Prognostic Score; GNRI, Geriatric Nutritional Risk Index; CONUT, Controlling nutritional status.

**Figure 4:**
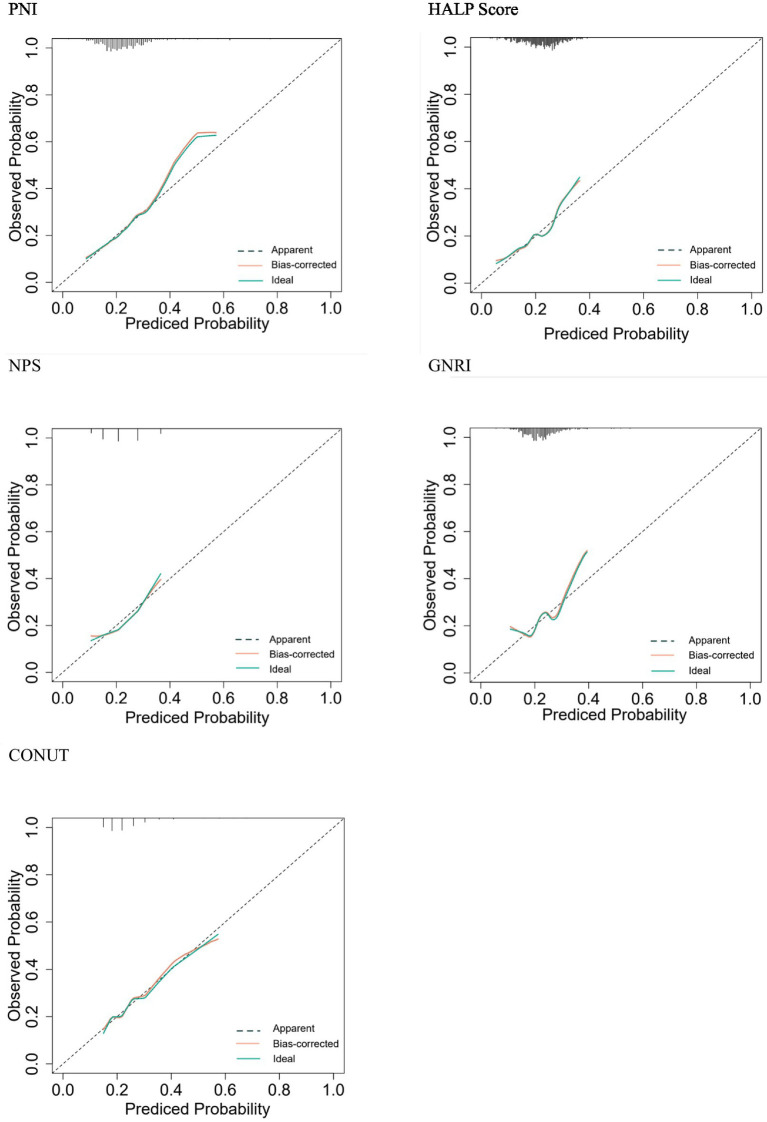
Standard curve.

**Figure 5:**
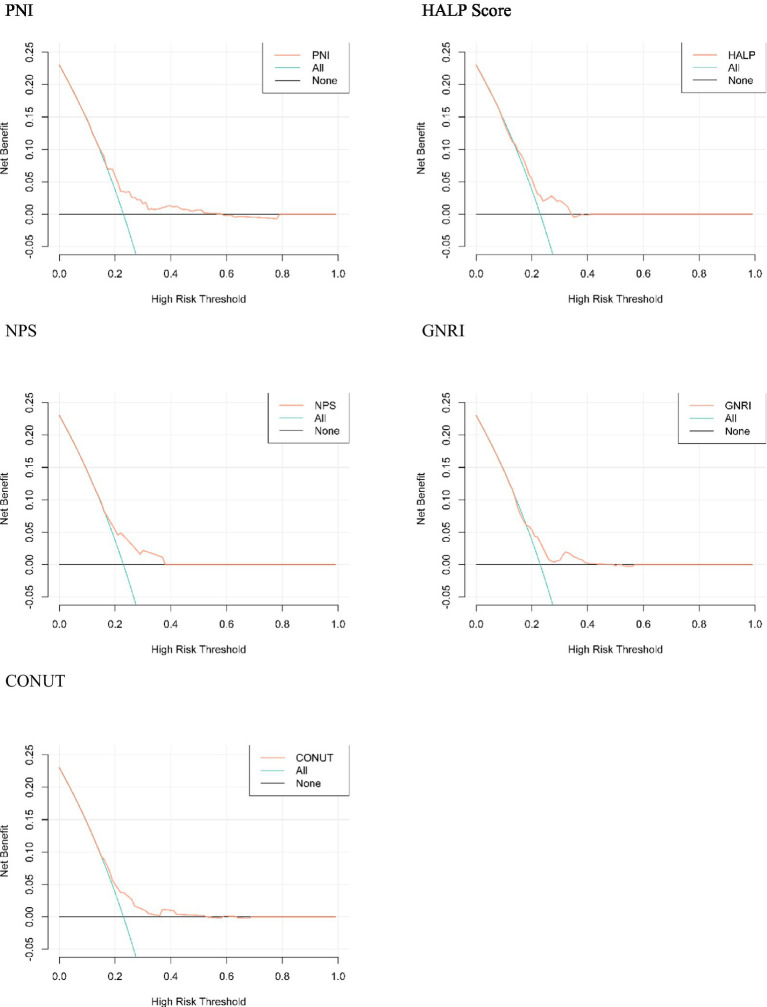
Decision analysis curve.

A prediction model was constructed using five nutritional assessment tools, including PNI, HALP Score, NPS, GNRI, and CONUT Score, to ascertain whether the prognosis of older adult patients with AIS would be poor within one year. The ‘regplot’ and ‘rmda’ packages in R software were employed to construct calibration curves and clinical decision curves, with the objective of determining the differentiation, calibration, and clinical impact of each of the five nutritional indicators in predicting whether or not a poor prognosis will occur within one year in older adult patients with AIS. The calibration curves and clinical decision curves were constructed with the objective of determining the degree of differentiation of the five nutritional indicators in predicting the occurrence of a poor prognosis in older adult AIS patients within one year. The horizontal coordinate of the calibration curve is the predicted probability of the model, and the vertical coordinate is the observed probability in the actual data. According to the calibration curves, the four predictive models constructed by PNI, HALP Score, NPS, and CONUT Score have good calibration ability (Figure 4); in the graph, there is a black line, a blue line, and a red line, the black line means that all people do not treat, then the net benefit of treatment is definitely 0. The blue line means that all people are treated, and then the value decreases as the threshold probability increases. The red line is a line plot of threshold probability versus net benefit for the decision model. Using the black and blue lines as reference lines, a model with a red line close to the reference line indicates no application, and one that is above the reference line over a large threshold interval indicates a better model. Figure 5 illustrates the decision analysis curve (DCA), which suggests that PNI at a threshold probability greater than 15% is a more favorable approach than implementing an intervention program for all patients in predicting the risk of a poor prognosis for older adult patients with AIS at 1 year. The net benefit of the predictive model is significantly higher than that of the all-or-none intervention.

## 4 Discussion

The objective of this study was to identify the most appropriate nutritional assessment tools for predicting the short-term prognosis of older adult patients with AIS from five nutritional assessment tools, including the PNI, HALP Score, NPS, GNRI, and CONUT Score. The following tools were considered: The findings of this study indicate that an elevated risk of malnutrition is associated with unfavorable clinical outcomes in older adult patients with AIS. Furthermore, the results demonstrate that PNI is an independent risk factor for poor prognosis in older adult patients with AIS and that PNI has a high predictive value for poor prognosis in older adult patients with AIS.

The process of aging is an irreversible biological phenomenon that is associated with a decline in the reserves of organ systems and a reduction in the ability of the body to maintain homeostasis (17). It can be reasonably deduced that older individuals are prone to developing nutritional issues as a consequence of the deterioration of their organ and bodily functions, the presence of underlying diseases, and their dietary habits. The occurrence of undernutrition has the potential to negatively impact the quality of life and disease outcomes in older individuals, leading to a reduction in their physical activity, impairment of their immune system, and a further decline in the functionality of their organs (18). The extant literature indicates that stroke patients with poor nutritional status tend to remain in the hospital for a longer period of time and exhibit more severe illness (19).

In this context, an initial nutritional assessment may be important for prognosis after stroke (20). However, there are currently no recognized nutritional screening tools, particularly for stroke patients. Tools employed for the purpose of screening for nutritional status, such as the Universal Screening Tool for Malnutrition or the Primary Nutritional Risk Index, necessitate the cooperation of patients in the completion of questionnaires or the reporting of recent loss of body mass. Furthermore, these nutritional assessment methods are arbitrary and subjective, necessitating comprehensive training by healthcare professionals or normal cognitive functioning on the part of the patient. It is therefore evident that these methods are not appropriate for screening all patients who have suffered an ischemic stroke. Some studies have demonstrated that biochemical indicators associated with nutritional status, such as total cholesterol (TC), serum albumin, transferrin, and prealbumin, are serum markers that exhibit inconsistent validity as determinants of a patient’s nutritional status. In contrast, five nutritional assessment tools, including PNI, HALP Score, NPS, GNRI, and CONUT Score, require only routine clinical blood test results and height and weight for rapid assessment, thus offering a convenient alternative to other, more complex methods (21–23).

Nutritional disorders are common in stroke patients and are negatively associated with poor clinical outcomes, both short- and long-term, with disability and death being the most critical issues (24). Studies have shown that lower serum albumin levels in stroke patients are associated with poorer outcomes. Due to age-related changes in body composition, such as sarcopenia, obesity, vertebral compression, and edema, body mass index (BMI) may be less accurate in the older adult population (25). Furthermore, it is crucial to acknowledge that the nutritional status of a patient during their hospitalization can be adversely affected by a number of factors, including a lack of adequate food intake, the presence of inflammatory complications, and the presence of comorbidities (26). It is therefore recommended that nutritional screening should form an integral part of the multidisciplinary care of stroke patients in the clinical setting. Previous studies have concentrated on the predictive value of PNI and CONUT Score in patients with AIS, focusing on short-term prognosis (2, 27, 28). The prognostic significance of malnutrition for long-term prognosis remains unclear. Zhang et al. reported that malnutrition on admission predicted functional recovery at 12 months in patients with AIS (29). Yuan et al. employed the CONUT Score and PNI indices to ascertain an association between malnutrition and long-term mortality in older adults who had experienced a first ischemic stroke (2). The results of this study are in accordance with those previously reported by Zhang et al., which indicated that malnutrition assessed at admission is a predictor of 12-month prognosis in patients with AIS.

In this study, we evaluated the nutritional status of older adult AIS patients using five nutritional assessment tools, including PNI, HALP Score, NPS, GNRI, and CONUT Score, and investigated the relationship between these assessments and poor prognosis. The primary findings were as follows: among the five nutritional assessment tools, the AUC value of PNI was greater than that of the remaining four nutritional assessment tools. The IDI and the NRI revealed that the incremental value of PNI in predicting risk was greater and that the risk of malpractice was higher in patients with low nutritional scores than in those with high nutritional scores (26). The standard and DCA curves demonstrated that the PNI exhibited excellent discrimination and calibration in predicting the occurrence of poor prognosis within one year in older adult patients with AIS.

Despite the existence of numerous screening tools for malnutrition, there is currently no consensus regarding the optimal screening tool for use in older adult patients with AIS. In light of the aforementioned findings, it is this author’s recommendation that the PNI score be employed, as it utilizes a mere two laboratory values and is readily calculable even in the absence of a dedicated automated calculator. It may be beneficial for these patients to participate in targeted secondary prevention programs and receive nutritional supplementation, with the aim of improving their prognosis.

The present study is limited in several ways. This is a single-center retrospective study with a relatively small number of patients, which introduces several limitations to the study. A comparison of the prognostic value of a nutritional screening tool with that of a more complex comprehensive nutritional assessment was not undertaken. Malnutrition is a complex phenomenon, particularly in the older adult, with a multitude of potential etiologies and contributing factors. The validity of a simple screening tool to assess nutritional status alone is questionable due to the absence of a comparison with comprehensive nutritional assessments, such as subjective global assessments and mini-nutritional assessments. Further validation of the results is required with larger samples and multi-center data. A comprehensive grasp of the interplay between nutritional indicators and adverse events in patients over the course of a year would facilitate a more precise evaluation of the correlation between these variables. We extend an invitation to other researchers and medical centers in different countries to contribute to the further development of this study.

## Data Availability

The original contributions presented in the study are included in the article/supplementary material, further inquiries can be directed to the corresponding authors.
